# Burnout and Its Association With Competence Among Dental Interns in China

**DOI:** 10.3389/fpsyg.2022.832606

**Published:** 2022-03-23

**Authors:** Yingjun Liu, Yi Song, Yong Jiang, Chuanbin Guo, Yongsheng Zhou, Tiejun Li, Wenshu Ge, Na An

**Affiliations:** ^1^Department of General Dentistry II, Peking University School and Hospital of Stomatology, National Center of Stomatology, National Clinical Research Center for Oral Diseases, National Engineering Research Center of Oral Biomaterial and Digital Medical Devices, Beijing, China; ^2^Institute of Child and Adolescent Health, School of Public Health, Peking University, Beijing, China; ^3^Department of Oral and Maxillofacial Surgery, Peking University School and Hospital of Stomatology, Beijing, China; ^4^Department of Prosthodontics, Peking University School and Hospital of Stomatology, Beijing, China; ^5^Department of Pathology, Peking University School and Hospital of Stomatology, Beijing, China

**Keywords:** self-evaluation, burnout, competency, self-stress, intern dentists

## Abstract

Intern physicians are generally more burdened by stress than the general population. This cross-sectional study aimed to evaluate the current situation regarding burnout and explore its association with the self-evaluation of competence among Chinese dental interns. A self-administered anonymous survey was conducted on 91 dental interns in the Peking University School of Stomatology, from August 2019 to June 2020. It consisted of a psychological stress questionnaire, including burnout and self-evaluation of clinical competence. The Wilcoxon signed rank test was used to determine the differences between self-evaluation scores of clinical competence. Results showed average scores for emotional exhaustion, depersonalization, and personal accomplishment of 22.22 ± 9.04, 8.16 ± 5.21, and 36.08 ± 7.76, respectively. Dental clinical technology was considered more useful than other clinical competencies, and there was a correlation between its importance and the stress caused by its deficiency (*r* = −0.201, *p* = 0.056). Significant associations were found between stress due to a lack of dental clinical technology and high emotional exhaustion (*r* = 0.273, *p* < 0.05). Burnout was common among the dental interns, which may be a valuable finding. Among the six different aspects of clinical competence, “dental clinical technology” represented the most stressful item. Strengthening pre-clinical training and promptly conducting targeted training in the early clinical process may be considered as decompression measures.

## Introduction

Burnout was first described in 1974 by Freudenberger, as a state of mental exhaustion due to a professional activity that fails to produce the expected expectations (Edu-Valsania et al., 2022). Most widely accepted definition of burnout is a psychological syndrome characterized by emotional exhaustion (EE), depersonalization (DP), and sense of reduced personal accomplishment (PA; Ahola and Hakanen, 2007). EE dimension manifests the exhaustion feeling caused by overwhelming work. It causes individuals to lack enough emotional energy to deal with work tasks. DP dimension is defined as a response of detachment, indifference, and unconcern toward the work being performed and/or the people who receive it. PA dimension is reflected in a lack of personal achievement and a negative professional self-evaluation (Edu-Valsania et al., 2022). Burnout is associated with increased risk for cardiovascular disease and developing musculoskeletal pain, depressive symptoms, and suicide (Salvagioni et al., 2017). Besides those physical and psychological consequences of individual, prospective effects of job burnout of occupational consequences include poor motivation and performance, reduction in the quality of services, as well as absenteeism.

Medical staff are prone to burnout due to their long work hours under high intensity and professional pressure. Studies have found a significant increase in the number of burnout cases among physicians compared to those in other careers, even after adjusting for work hours and other factors (Shanafelt et al., 2012a; Dyrbye et al., 2014). Noteworthy prevalence of burnout (near or more than 50%) had been documented of both physicians-in-training and practicing physicians in the United States (West et al., 2018). Burnout among physicians have negative impact both on their own health and the quality of clinical care they provide. The consequences of burnout include the risk of medical errors, reduced productivity and job satisfaction, higher physician turnover, and adverse effects on patient safety (Slavin, 2019; Zhou et al., 2020).

Investigations on the origins of physician burnout reported that the phenomenon may start to manifest as early as in medical school (Ishak et al., 2013). A study about United States physician at different career stages showed early career physicians (in practice 10 years or less) had the highest rates of DP (Dyrbye et al., 2014). Numerous factors may cause stress to early career students and interns, including workload, academic factors, complicated and irreversible interventions, conflicts with patients, and limited time to perform and finish the planned treatment (Elani et al., 2014 Basudan et al., 2017).

The prevalence of burnout may vary according to specialty. Dental students may experience greater stress levels than medical students and suffer from greater burnout. Therefore, it is important and meaningful to identify burnout and investigate its influencing factors among dental intern. Compare with medical students, dental intern students suffer EE and DP more frequently (Jimenez-Ortiz et al., 2019). Dental interns face requires for both theoretical and clinical knowledge, as well as face-to-face practice and daily patient interaction (Basudan et al., 2017). Different from other majors, dental interns carry out many meticulous clinical operations in the early stage of their career. These operations include multiple complex steps, some of which are irreversible. It is not easy to successfully complete them and achieve satisfactory results within the specified time. According to ADEA, competence essential for the general dentist to begin independent, unsupervised dental practice includes Patient care, Health Promotion, Professionalism, Critical Thinking, Communication and Interpersonal Skills, Practice Management, and informatics (Albino et al., 2008). The lack of clinical competence may be one of the causes of burnout because interns may be under greater psychological pressure due to a lack of clinical experience or ability in the initial stages of their career.

Some studies have focused on the burnout of dental interns, but major of them focus on the relationship between stress and burnout. There is a lack of research on the association between clinical competence deficiency and burnout among dental interns. Recognizing deficiencies and promptly conducting targeted training are important components of medical education. This study assessed the interns’ self-evaluation of clinical competence, trying to find targeted measures for decompression.

The current studies were mainly conducted in Europe and the Unit States, and studies on the burnout of Chinese dental interns are limited. Therefore, we carried out this study for dental interns, aimed to evaluate the current situation regarding burnout and explore its association with the self-evaluation of competence among dental interns in a hospital of stomatology in Beijing, China. This study indicates that burnout among dental interns is common and should not be ignored.

## Materials and Methods

### Study Design and Participants

This study consisted of a cluster sampling of all dental interns in a certain department of Peking University School of Stomatology, which is ranked as the best school of stomatology in China. This department provides clinical practice sites and clinical education for dental interns including 5-year system students, 8-year system students, professional master graduate student, and trained general dentistry registrars. The survey was conducted from August 2019 to June 2020.

This study protocol was approved by the Medical Ethics Committee of Peking University School and Hospital of Stomatology.

A questionnaire was used to conduct a self-administered anonymous survey. The questionnaire included four parts: general information, stress and physical health self-evaluation, burnout [Maslach Burnout Inventory-Human Service Survey (MBI-HSS)], and self-evaluation of clinical competence. During the survey, all dental interns who have clinical practice in this department got paper questionnaire at the end of the practice period. Participants voluntarily return questionnaire. A total of 91 valid questionnaires were collected (participation rate was 90.7%).

### Stress and Physical Health Self-Evaluation

The items related to the stress and decompression self-evaluations were assessed on a 5-point Likert scale. Respondents were asked to evaluate the clinical psychological pressure and scientific research-related academic pressure in the past month using numbers from 1 to 5 (1 = *no pressure*, 2 = *less pressure*, 3 = *moderate pressure*, 4 = *greater pressure*, and 5 = *very stressed*).

As for physical health evaluation, the questionnaire raised the following specific questions: (1) How many times have you participated in decompression training in the past year? (2) Which of the following descriptions is closer to your self-evaluation of physical health status during the past month: excellent, good, average, or poor?

In China, WeChat is a widely used app that can record daily walking steps with the permission of users. The highest and lowest number of interns’ daily walking steps recorded in the past 7 days were collected. These numbers were used as a measurement index to evaluate their daily exercise status.

### Burnout

The survey tool for burnout was the internationally used MBI-HSS, which is the most widely accepted standard for burnout assessment of healthcare professionals (West et al., 2018). In previous studies on dental staff, the Chinese version of the MBI-HSS has reported acceptable Cronbach’s α coefficients (0.68–0.89; Lee et al., 2019). The scale includes 22 questions on three dimensions. Each item is ranked on a 7-point Likert scale ranging from 0 (*never*) to 6 (*every day*), according to the degree of compliance. The EE dimension has nine items, the sum score ranges from 0 to 54, and mild, moderate, and severe burnout are defined as sum scores ≤16, 16–27, and > 27, respectively. The DP dimension has five items, the sum score ranges from 0 to 30, and mild, moderate, and severe burnout are defined as sum scores ≤6, 6–13, and > 13, respectively. The PA dimension has eight items, the sum score ranges from 0 to 48, with mild, moderate, and severe burnout defined as sum scores ≥39, 31–39, and < 31, respectively.

### Self-Evaluation of Competence

The self-evaluation of competence included six domains: Patient care, Health Promotion, Professionalism, Critical Thinking, Communication and Interpersonal Skills, and Practice Management and informatics. The definitions of competencies were explained to respondents according to the statements approved by the 2008 ADEA House of Delegates (Albino et al., 2008). There were five items for each competence: (i) I think it is necessary to get this competence (Usefulness); (ii) I have mastered this competence well (Mastery); (iii) the lack of competence caused psychological pressure (Stress); (iv) the competence is difficult to obtain (Difficulty); and (v) I spend a lot of time acquiring this competence (Time spent). According to the degree of compliance, a 5-point Likert scale ranging from 0 (*very inconsistent*) to 4 (*very consistent*) was used.

### Statistical Analyses

All cases were entered into the computer with EpiData 3.1, and data were entered twice to reduce errors. If it was found that the results of the two entries were different or the results had abnormal values, the original questionnaire was retrieved for verification and correction.

A statistical analysis was performed using SPSS (version 24.0; IBM, Armonk, New York). All study variables were analyzed using descriptive statistics. Quantitative variables were described as mean ± standard error. Categorical variables were expressed as frequencies (n) and percentages (%). Independent sample t-tests and one-way ANOVA were used to compare the differences in burnout between genders, identity, and education level. The scores of competence self-evaluation which not correspond to normal distribution were analyzed using Related samples Wilcoxon signed rank test. Pearson’s correlation analysis was used to explore the correlations between burnout and competence. *p* < 0.05 was considered statistically significant with a Confidence Interval of 95%.

## Results

### Demographics

Table 1 shows that 91 general dentistry registrars and undergraduate students participated in this study. The age range was 21–36 years, with a median of 23 years. The median of clinical practice was 1.5 years.

**Table 1:** Demographic characteristics of the sample.

		Total	Female	Male	
**Identity**	5-year system students	26 (28.6%)	16	10	
	8-year system students	31 (34.1%)	23	8	
	Professional master graduate student	13 (14.3%)	9	4	
	Trained general dentistry registrars	21 (23.1%)	17	4	
**Educational level**	Bachelor	69 (75.8%)	48	21	
	Master	9 (9.9%)	6	3	
	Doctor	13 (14.3%)	11	2	
**Total**		91 (100.0)	65 (71.4%)	26 (28.6%)

### Self-Stress and Physical Health Evaluation

As shown in Table 2, the average value of self-evaluation of scientific research pressure for the past month was 2.36 ± 1.28, and the average value of self-evaluation of clinical pressure for the past month was 2.41 ± 0.87. In the past year, 86.8% of the respondents had not participated in any psychological decompression training, 8.8% had participated once, 1.1% had participated twice, and 3.3% had participated more than twice. The self-evaluation of their physical health status for the past month was excellent for 20.9%, good for 41.8%, average for 33%, and poor for 4.4%. The average sleep time in the past week was 6.6 h. The highest number of daily steps was 11823.86 ± 4177.11, and the lowest number of steps was 2860.49 ± 2617.57.

**Table 2:** Self-stress and physical health evaluation.

	Total	Female	Male
**Score of self-stress evaluation for the past month**			
Scientific research	2.36 ± 1.28	2.32 ± 1.06	2.46 ± 1.73	
clinical	2.41 ± 0.87	2.31 ± 0.81	2.65 ± 0.98
**Self-evaluation of physical health status for the past month**			
Excellent	19 (20.9)	11	8
Good	38 (41.8)	29	9
Average	30 (33%)	22	8
Poor	4 (4.4%)	3	1
**Psychological decompression training participation in the past year**			
none	79 (86.8%)	56	23
Once	8 (8.8%)	6	2
Twice	1 (1.1%)	0	1
More than twice	3 (3.3%)	3	0
**Times of exercise for more than 60 min in the past week**			
0	38 (41.8%)	29	9
1	20 (22%)	13	7
2	17 (15.4%)	12	5
3	6 (6.6%)	4	2
4	7 (7.7%)	6	1
5	1 (1.1%)	1	0
6	0 (0%)	0	0
7	1 (1.1%)	0	1
8	1 (1.1%)	0	1
**Number of daily steps in the past week**			
Highest	11823.86 ± 4261.87 4,551 ~ 23,113	11931.13 ± 4396.09 4,551 ~ 23,113	11542.90 ± 3624.53 5,910 ~ 20,000
Lowest	2860.49 ± 2617.57 100 ~ 10,000	2898.73 ± 2723.27 100 ~ 10,000	2760.33 ± 2378.11 100 ~ 8,000

### Burnout Among Dental Interns

The EE score was 22.22 ± 9.04, ranging from 1 to 41, the DP score was 8.16 ± 5.21, ranging from 0 to 39, and the PA score was 36.08 ± 7.76, ranging from 0 to 48. The results of this study are similar to those of a known survey of burnout among medical practitioners (Table 3).

**Table 3:** Maslach Burnout Inventory subscale scores for intern dentists.

	Mild (%)	Moderate (%)	Severe (%)	Average of Participating intern dentists
**EE**	24 (26.37%)	38 (41.76%)	29 (31.87%)	22.22 ± 9.04
**DP**	36 (39.56%)	39 (42.86%)	16 (17.58%)	8.16 ± 5.21
**PA**	36 (39.56%)	35 (38.46%)	20 (21.98%)	36.08 ± 7.76

EE, emotional exhaustion; DP, depersonalization; and PA, personal accomplishment.

Table 4 presents the Maslach Burnout Inventory subscale scores according to demographic variables. As the table illustrates, there are no significant differences in the means between different educational levels. Female participants had higher EE scores and lower PA scores than male participants, which implies a higher prevalence of burnout among female dental interns than among male dental interns.

**Table 4:** Maslach Burnout Inventory subscale scores according to sociodemographic variables.

Variables	*n*	EE	DP	PA
**Gender**				
Female	65	25.27 ± 7.26^a^	8.46 ± 4.51	34.08 ± 6.86^b^
Male	26	21.00 ± 9.44^b^	8.05 ± 6.68	36.88 ± 7.99^a^
**Identity**				
5-year system students	26	23.88 ± 8.33	9.94 ± 6.23^c^	36 ± 6.63
8-year system students	31	23.33 ± 9.18	9.83 ± 7.50^c^	37.29 ± 6.07
Professional master graduate student	13	19.38 ± 8.53	4.46 ± 2.50^d^	36.69 ± 6.52
Trained general dentistry registrars	21	20.10 ± 9.99	5.76 ± 3.82^d^	34.43 ± 11.28
**Educational level**				
Bachelor	69	22.77 ± 8.88	8.97 ± 6.52	36.46 ± 6.94
Master	9	22.11 ± 8.15	5.00 ± 2.29	35.00 ± 6.12
Doctor	13	19.38 ± 10.54	6.08 ± 4.55	34.77 ± 12.22

a is significantly higher than

b *p* < 0.05 (Independent sample *t*-tests)

c is significantly higher than d, *p* < 0.05 (One-way ANOVA).

### Self-Evaluation of Competence Among Dental Interns

As shown in Table 5, patient care was considered to be more useful than other competencies. In fact, there is a correlation between the importance of patient care and the stress caused by its deficiency (*r* = −0.201, *p* = 0.056, Pearson correlation analysis), as it took longer to acquire compared with other competencies. However, the dental interns thought that mastering this competence was similar to others in their self-evaluation.

**Table 5:** Self-evaluation of clinical competence among intern dentists.

	Patient Care	Health Promotion	Professionalism	Critical Thinking	Communication and Interpersonal Skills	Practice Management and Informatics
Usefulness	3.84 ± 0.70	3.42 ± 0.94^*^	3.11 ± 1.19^*^	3.34 ± 1.04^*^	3.58 ± 0.98	3.01 ± 1.18^*^
Mastery	2.18 ± 0.72	2.26 ± 0.79	2.32 ± 0.79	2.32 ± 1.10^&^	2.62 ± 0.74^&^	1.92 ± 0.87
Stress	2.22 ± 1.01	1.41 ± 0.92^#^	1.05 ± 0.99^#^	1.60 ± 1.02^#^	1.57 ± 1.00^#^	1.30 ± 0.92^#^
Difficulty	2.31 ± 0.99	1.74 ± 0.85	1.57 ± 1.08	2.44 ± 1.25	2.00 ± 1.06	1.90 ± 0.94
Time spent	3.21 ± 0.84	2.16 ± 0.96^@^	1.95 ± 1.11^@^	2.58 ± 0.96^@^	2.21 ± 1.01^@^	2.03 ± 0.94^@^

* Compared with usefulness of dental clinical technology, *p* < 0.05 (related samples Wilcoxon signed rank test).

& Compared with mastery of usefulness of dental clinical technology, *p* < 0.05 (related samples Wilcoxon signed rank test).

# Compared with the degree of stress caused by the lack of dental clinical technology, *p* < 0.05 (related samples Wilcoxon signed rank test).

@ Compared with time spent on dental clinical technology, *p* < 0.05 (related samples Wilcoxon signed rank test).

There were significant correlations between the stress caused by the lack of patient care competence and a high EE score (*r* = 0.273, *p* < 0.05, Pearson correlation analysis, Table 6).

**Table 6:** Pearson correlation between burnout and dental clinical technology.

Pearson correlation	Usefulness	Mastery	Stress	Difficulty	Time spent
EE	−0.227^*^	0.271^**^	0.273^**^	0.266^*^	−0.185
DP	−0.009	−0.063	0.004	0.182	0.041
PA	0.203	−0.188	−0.182	−0.020	0.289^**^

* signifies that correlation is significant at the 0.05 level.

** signifies that correlation is significant at the 0.01 level (Pearson correlation analysis, two tailed).

## Discussion

Physician burnout is increasingly prevalent and may become a public health crisis because it is not only associated with their own negative attitude but is also related to negative consequences on patient care, the physician workforce, and healthcare system costs (Dyrbye et al., 2014). Some studies supported the view that burnout may begin manifesting as early as medical school (Santen et al., 2010; Bughi et al., 2017). It is of great significance for the public health to detect the burnout of medical staff in early stage and intervene in time.

Burnout among dental interns is common and this fact that must be addressed. While this study used diverse samples from different cultural contexts, similar conclusions regarding burnout were reached. Our findings were consistent with a recently published mate-analysis study regarding professional burnout rates among medical students, measured with the MBI-HSS, with weighted mean values of 22.93 ± 10.25 for EE, 8.88 ± 5.64 for DP, and 35.11 ± 8.03 for PA (Erschens et al., 2019). The value of DP was 7.12 ± 5.22 in the early published normative data (Willcock et al., 2004), and our findings (8.16 ± 5.21) are between the results of the early studies and recent reviews. The results of this study do not support the higher level of burnout of dental interns compared with other medical majors on a world-wide scale. Due to the few available studies, it is difficult to compare whether Chinese dental interns suffer more serious burnout than other Chinese medical students. The only available study on burnout of Chinese medical students was conducted at Sun Yat-sen University in 2018 used MBI-Student Survey (MBI-SS) as survey tool. In this early study, there was no significant difference between 374 students majored in clinical medicine and 79 students majored in other disciplines (basic medicine, forensic medicine, public health, nursing, and dentistry) in term of high burnout risk (Liu et al., 2018).

Current research shows that female dental interns seem to suffer from a more serious occupational burnout, which confirms some previous results on other majors, such as surgeons (Shanafelt et al., 2012b) and physicians (McMurray et al., 2000) in the United States. A greater prevalence of burnout among women was found in a study of 307 Jordanian dental students (Badran et al., 2010). However, some previous studies reached the opposite conclusion. An early study in 2003 among Dutch dentists showed that men who reported higher levels of DP tended to work 7.5 h more per week than women (De Brake et al., 2003). A review included 33 papers identified male gender was one of most prevalent and significant factors associated with burnout in dentistry (Singh et al., 2016). However, results from different countries may be influenced by various healthcare systems, cultures, and populations, which may reduce the external validity of the findings. A multi-country study among 5,104 dental students from 14 participating countries confirmed that the country itself plays a significant role as an independent variable (Alhajj et al., 2018). Another possible interpretation for these conflicting results is that gender plays a different role in different stages of a dentist’s career. The protective effect of the male gender toward burnout at the student level may disappear as the dentist’s career progresses (Singh et al., 2016).

This shows the need for actions and practices that allow intervening in such a reality, making this training period as less stressful as possible, contributing to the mental health of the trainees. Identifying the challenges faced by trainees at different levels and across different specialties may assist in improving the effective tailoring of interventions (Busireddy et al., 2017). According to our study, “patient care” and “dental clinical technology” were the most stressful items for dental interns. This suggests that medical educators should strengthen pre-clinical training and promptly conduct targeted training in the early clinical process.

Our results from the evaluation of 91 Chinese dental interns were in line with the results of previous investigations under different cultural contexts, thus indicating that burnout was common among them and should be considered important. Female participants suffered more from burnout in the EE and PA dimensions than male participants. Among the six different aspects of clinical competence, “patient care” and “dental clinical technology” were the most stressful items for dental interns.

## Limitations and Proposals

This study has several limitations: First, the generalizability of these results was restricted, as the study involved only 91 interns for specific department in 1 year. Our team will continue this survey in a larger sample population form different department and hospital to expand the sample size and compare the burnout in different periods. Second, this study also investigated the life habits of intern dentist, such as daily walking steps and sleep time, but it is difficult to make a comparison with similar population because the relevant studies on the issue in China have not been reported. It is expected that more effective analysis can be carried out after expanding the sample size in the future.

## Data Availability Statement

The raw data supporting the conclusions of this article will be made available by the authors, without undue reservation.

## Ethics Statement

The studies involving human participants were reviewed and approved by Ethics committee of Peking University School of Stomatology. Participation of the study objects were entirely voluntary and completely anonymous. The participants provided their oral informed consent to participate in this study.

## Author Contributions

YS and NA designed the research framework. YL implemented the research, provided the statistical analysis, and wrote the manuscript. YJ, CG, YZ, TL, and WG revised the manuscript. All authors approved the final version of manuscript for publication.

## Funding

This research was funded by the Education Project, Peking University School and Hospital of Stomatology (grant number 2019SZ04) and Education Research Project of Peking University Health Science Center in 2019 (grant number 2019ZP23).

## Conflict of Interest

The authors declare that the research was conducted in the absence of any commercial or financial relationships that could be construed as a potential conflict of interest.

## Publisher’s Note

All claims expressed in this article are solely those of the authors and do not necessarily represent those of their affiliated organizations, or those of the publisher, the editors and the reviewers. Any product that may be evaluated in this article, or claim that may be made by its manufacturer, is not guaranteed or endorsed by the publisher.
